# Case Report: *MDM4* Amplified in a Thymoma Patient With Autoimmune Enteropathy and Myocarditis

**DOI:** 10.3389/fendo.2021.661316

**Published:** 2021-05-13

**Authors:** Xin Du, Lei Yu, Fei Li, Zhen Yu, Xing-guo Yang, Yu-xuan Jiang, Xin-tao Yu

**Affiliations:** Department of Thoracic Surgery, Beijing Tongren Hospital, Capital Medical University, Beijing, China

**Keywords:** thymoma, autoimmune enteropathy, autoimmune myocarditis, *MDM4*, whole exome sequencing, immunohistochemistry, drug sensitivity test

## Abstract

**Introduction:**

Thymoma is a type of mediastinal malignant tumors which always associated with autoimmune diseases. Although surgery is the predominant treatment method for thymoma, the pathogenesis of thymoma and thymoma-associated autoimmune diseases is still unknown. However, the case study here provided a possible pathogenesis and treatment to cure the thymoma with autoimmune enteropathy and myocarditis.

**Case presentation:**

A thymoma case with autoimmune enteropathy and myocarditis undergoing surgery was reported. The symptoms and laboratory results of the patient had dramatically fluctuated after tumor resection and gradually alleviated. The whole exome sequencing found *MDM4* amplified in tumor cells. Immunohistochemistry indicated that thymoma cells were positive for MDM4. The result of drug sensitivity tests showed thymoma cells were highly sensitive to Nutlin-3a.

**Conclusion:**

*MDM4* could play an important role in the pathogenesis of this thymoma case with autoimmune enteropathy and myocarditis. This discovery may provide a novel idea of pathogenesis and treatment for thymoma and autoimmune diseases.

## Introduction

Although thymoma is the most common primary tumor of the anterior mediastinum (1), the annual incidence of the case is only 1.4/1,000,000 (2). More than one third of thymoma patients are associated with some kinds of paraneoplastic syndromes (3), such as myasthenia gravis (MG), pure red cell aplasia (PRCA), hypogammaglobulinemia, etc (4, 5). Some paraneoplastic syndromes may gradually alleviate after tumor resection. But up to now, the pathogenesis of thymoma and thymoma-associated autoimmune diseases has still not completely well explained. In fact, it is very important to understand the relationship between thymoma and its related autoimmune diseases, and potential abnormal molecule. In this way, we can go a step further towards the completely revealing the pathogenesis of both thymoma and autoimmune diseases. Here, we reported a thymoma case with autoimmune enteropathy and myocarditis.

## Case Presentation

### Case History

A 22-year-old young man presented with diarrhea for nearly 1 year and diagnosed of thymoma for 4 months. He is a Chinese college student, with no medical history. He denied any tobacco, alcohol, drug and family history.

The patient presented with diarrhea by sudden, did not have any recognizable precipitating factors. The frequency was 6 to 8 watery stools daily with tenesmus. There was no abdominal pain, hematochezia, or melena. The conventional antidiarrheal drugs and gluten-free diet were ineffective.

**Figure 1:**
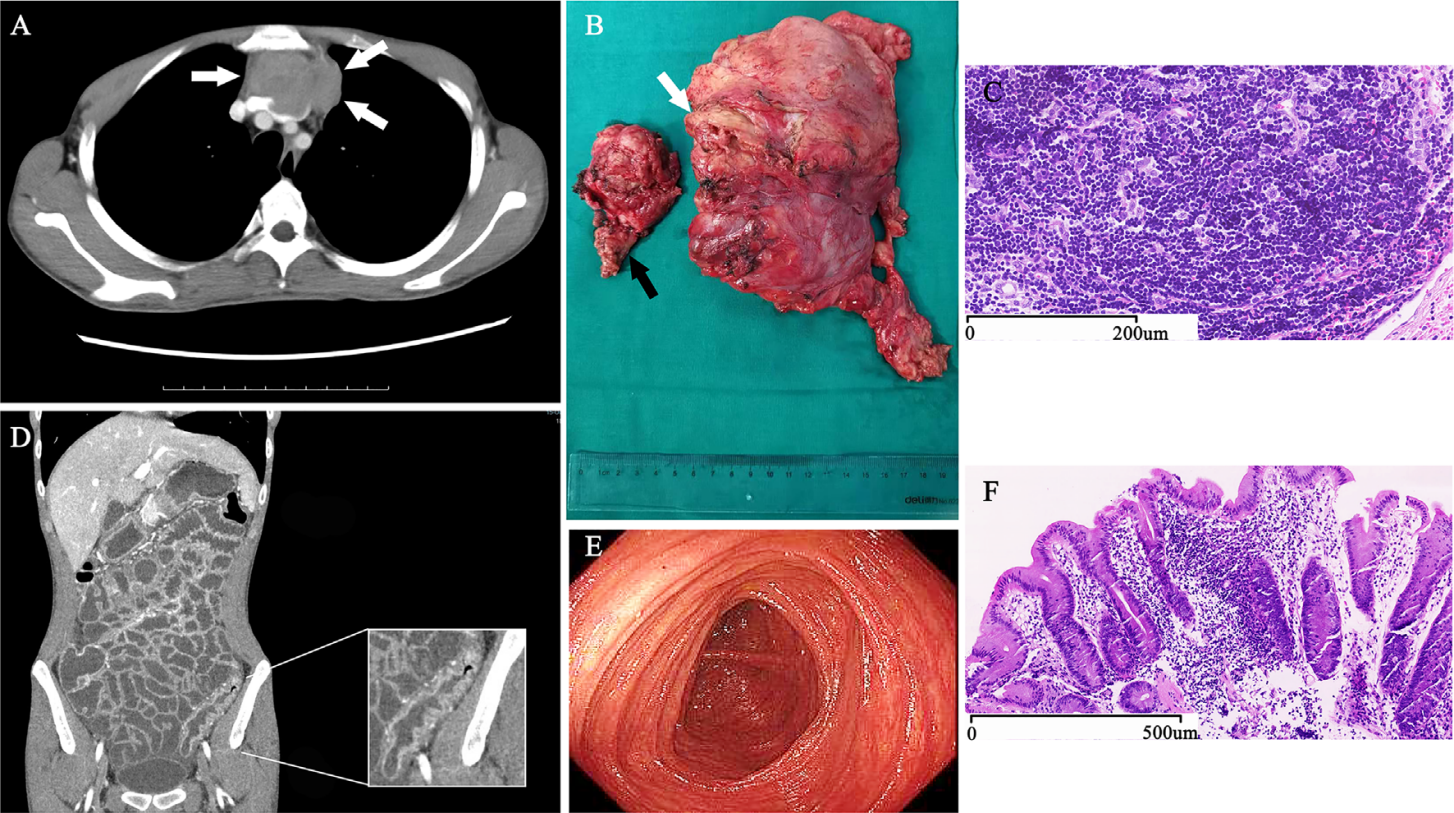
Diagnostic Evidence of Thymoma with Autoimmune Enteropathy and Myocarditis. An axial, CT contrast-enhanced scan of the chest **(A)** shows a mediastinal mass (arrows) invading into the left anonymous vein. During surgery, we found that not only the left anonymous vein (white arrow) but also the anterior segment of left-upper lobe (black arrow) were involved by the thymoma **(B)**. The pathological sections of tumor stained with hematoxylin and eosin shows typical B1 thymoma **(C)**. A coronal, CT contrast-enhanced scan of the abdomen and pelvis **(D)** shows the typical thickening and high-density of the colon wall. The enteroscopy **(E)** showed the sigmoid colonic mucosa was markedly thinner than normal, and the colon mucosal biopsies **(F)** detected the colonic mucosa with chronic inflammation, mild lymphocytosis, and extensive loss of goblet cells.

Four months ago, because of cough and shortness of breath, he underwent a chest computed tomographic (CT) examination, revealing a huge anterior mediastinal mass that measured 7.2 cm craniocaudally, 6.7 cm transversely, and 4 cm anteroposteriorly, as well as invading into the left anonymous vein (**Figure 1A**). 18F-flurodeoxyglucose positron emission tomography/CT showed the mediastinal mass was malignant, and no other malignant lesion was observed. Pathological examination by a core-needle biopsy specimen confirmed the diagnosis of thymoma. The abdomen and pelvis CT film showed the diffuse wall thickening of the colon (**Figure 1D**). The enteroscopy showed the small intestinal villus atrophy, and the sigmoid colonic mucosa was markedly thinner than normal (**Figure 1E**). The pathological examination confirmed the diagnosis of autoimmune enteropathy (6) (**Figure 1F**). In addition, the anti-intestinal goblet antibody, anti-centromere protein B antibody were also positive. Flow cytometric evaluation of lymphocyte subsets showed: CD3, 2266/µL, (reference range, 1185–1,901); CD4+, 782/µL, (reference range, 561–1,137); CD8+, 1334/µL, (reference range, 404–754). Assay of serum immunoglobulin levels showed: IgG levels higher than normal, while IgM, IgE, and IgD are normal.

**Figure 2:**
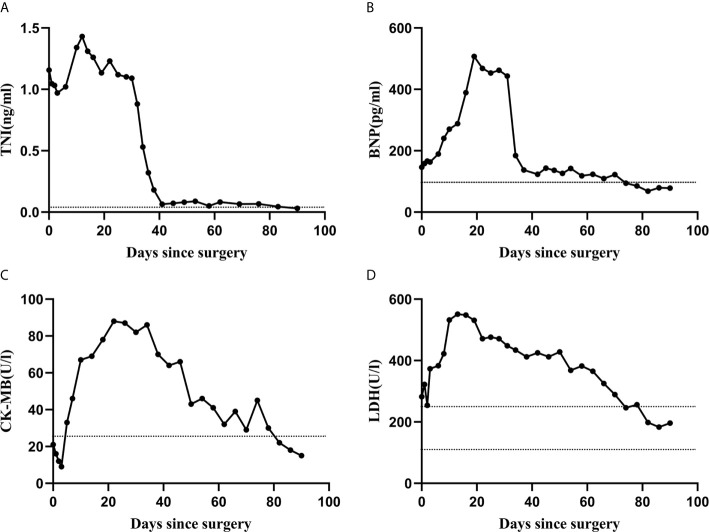
The trends of laboratory test results. The levels of TNI, BNP, LDH, and CK-MB were mildly higher than normal before surgery, significantly increased within 10 days after tumor resection, and 3 months later, returned to be normal **(A**–**D)**. Horizontal dashed black lines in each graph designate the upper and lower limits of the normal range for each variable, as determined by the Beijing Tongren Hospital Clinical Hematology Laboratory.

Considered that autoimmune enteropathy might be associated with thymoma, the patient was admitted to Beijing Tongren Hospital for tumor resection. On physical examination, the patient was severely malnourished and the vital signs were normal. Auscultation of the chest revealed scattered rales. The abdomen was soft, non-tender, and the bowel sound was exaggerated. The remainder of the physical examination was normal. On laboratory examination, levels of troponin I (TNI), B-type natriuretic peptide (BNP), creatine kinase-MB (CK-MB), and lactate dehydrogenase (LDH) were mildly higher than normal (**Figures 2A–D**). The patient was positive for anti–RO-52, anti-titin, and anti-myocardial antibody, negative for anti-body and DNA of cytomegalovirus, and suspected clinically of having autoimmune myocarditisr (7).

The oxygen saturation, renal-function and thyroid-function tests were normal; fecal occult blood tests (FOBT), hanging drop test, parasitological test, clostridium difficile toxin of stool were negative. The other laboratory results were shown in **Table 1**.

**Table 1:** Laboratory data.

Variable	Pre-operation	Post-operation				Reference
		3 days	10 days	6 weeks	3 months	Range*
Erythrocyte sedimentation rate (mm/h)	8	21	37	32	6	0–15
Alanine aminotransferase (U/l)	89	56	125	171	48	90–50
Aspartate aminotransferase (U/l)	72	64	102	97	52	15–40
Alkaline phosphatase (U/l)	550	291	746	466	167	45–125
γ-glutamyltransferase (U/l)	711	258	852	556	265	10–60
Creatine Kinase (U/l)	129	241	387	168	57	50–310
Anti-nuclear antibody	1:160	/	1:320	/	1:80	<1:40
Anti-intestinal goblet antibody	1:20	/	1:20	/	(-)	<1:10
Anti-myocardial antibody	1:160	/	1:320	/	1:160	<1:100
Anti-centromere protein B antibody (IU/ml)	116	/	124	/	12	<15
Anti‐Ro52 antibody(IU/ml)	128	/	169	/	78	<15
Titin-Ab	(++)	/	(+++)	/	(+)	(-)
IgG (mg/dl)	1815	1143	847	763	704	700–1600
Differential count (%)						
B lymphocytes (CD3-CD19+)	7.6	7.4	7.2	2.1	15.3	6–26
T lymphocytes (CD3+)	89.5	85.2	89.2	86.1	80.9	55–84
CD4+/CD8+ T-lymphocytes ratio	0.59	0.59	0.53	0.68	0.69	0.71–2.78

*Reference values are affected by many variables, including the patient population and the laboratory tests used in this case. The ranges adopted at the Beijing Tongren Hospital are age-adjusted and for patients who are not pregnant and do not have medical conditions that could affect the results. So they might not be suitable for all patients in other medical center.

Thymoma, left anonymous vein and involved anterior segment of the left-upper lobe were completely resected *via* median sternotomy (**Figure 1B**). The surgical procedure was smooth. The amount of intraoperative bleeding was 200 ml and no transfusion.

### Clinical Data and Specimen Collection

The patient and his parents provided written informed consent for their participation in the study, specimen collection, and publication of the results. During the consenting process, risks and benefits of research-based whole exome sequencing (WES), immunohistochemistry (IHC) and drug sensitivity testing (DST) were explained.

### Whole Exome Sequencing (WES) Analysis

Genomic DNA was isolated from peripheral blood and tumor tissue, detected among the whole exome of more than 20,000 exons by SureSlect Human All Exon V6 of Agilent Technologies, Inc.

### Histologic and Immunohistochemical Testing

The paraffin-embedded thymoma tissues were cut into 4 µm of sections, stained with hematoxylin and eosin (H&E) and analyzed by immunohistochemistry (IHC).

### Drug Sensitivity Testing

The collagen gel droplet embedded culture-drug sensitivity test (CD-DST) was performed as we described previously (8, 9). A sample of thymoma was collected from the fresh specimen during surgery. After 5–7 days’ growth, the colonies of thymoma cells were cultured in collagen gel droplets with Nutlin-3a (1.0 μg/ml) and analyzed by the image analysis method (the software Primage 1.0.6.3). The drug sensitivity was measured by the size of colonies. By measuring the size of colonies, drug sensitivity was tested.

## Results

The postoperative pathological diagnosis indicated B1 thymoma, and the tumor margin was clean and no tumor cell infiltration was observed (**Figure 1C**). Because it invaded into the left anonymous vein, the tumor was diagnosis as stage III according to the Masaoka stage. The diarrhea improved significantly within 3 days after operation. The patient self-reported that the appetite and mental conditions improved significantly postoperatively. And then, the patient was discharged. However, the symptom of diarrhea reappeared 5 days after surgery. The patient presented with dyspnea and respiratory muscle weakness 10 days after surgery. Within 2 weeks, these symptoms rapidly worsened. Initial vital signs at emergency department were as follows: blood pressure, 106/65 mm Hg; heart rate, 120 to 130 beats/min; respiratory rate, 25 to 33/min; body temperature, 36.3°C and blood oxygen saturation, 93% to 95% (33% FiO_2_). Echocardiography demonstrated diffuse myocardial wall hypokinesis with reduced left ventricular ejection fraction (LVEF) less than 35%. The ECG showed sinus tachycardia and occasional ventricular premature beat. Levels of TNI, BNP, CK-MB and LDH drastically increased within 10 days after surgery (**Figures 2A–D**). Meanwhile, percentage of CD3 increased, and CD4+/CD8+ decreased (shown in **Table 1**). Tracheal intubation with ventilator was immediately used for respiratory support. Edrophonium testing was negative. The acetylcholine receptor (AChR) antibody and muscle-specific receptor tyrosine kinase (MuSK) antibody were negative in the serum analyzed by radioimmunoassay. No decrement were observed in repetitive nerve stimulation (RNS) of facial nerve, accessory nerve and ulnar nerve at 3 Hz and 5 Hz.

The patient was treated with intravenous methylprednisolone 500 mg daily for 3 days, 80 mg daily for 2 weeks, and then, oral methylprednisolone 28 mg once a day. The dose of methylprednisolone reduced 4 mg per 2 weeks. After the medical treatment, the symptom of watery stools improved gradually, and 3 months later, return to be normal. The patient could also wean off the ventilator and the echocardiography showed LVEF was 55%. Levels of TNI, BNP, CK-MB and LDH gradually decreased and returned to be normal (**Figures 2A–D**), and the same trend was observed in the percentage of CD3 and CD4+/CD8+ (shown in **Table 1**). Chest CT examination at 2 weeks, 1 month, 2 months, and 3 months after surgery showed no signs of tumor recurrence or metastasis. The patient self-reported that diarrhea, dyspnea and muscle weakness were completely relieved 3 months after the operation.

**Figure 3:**
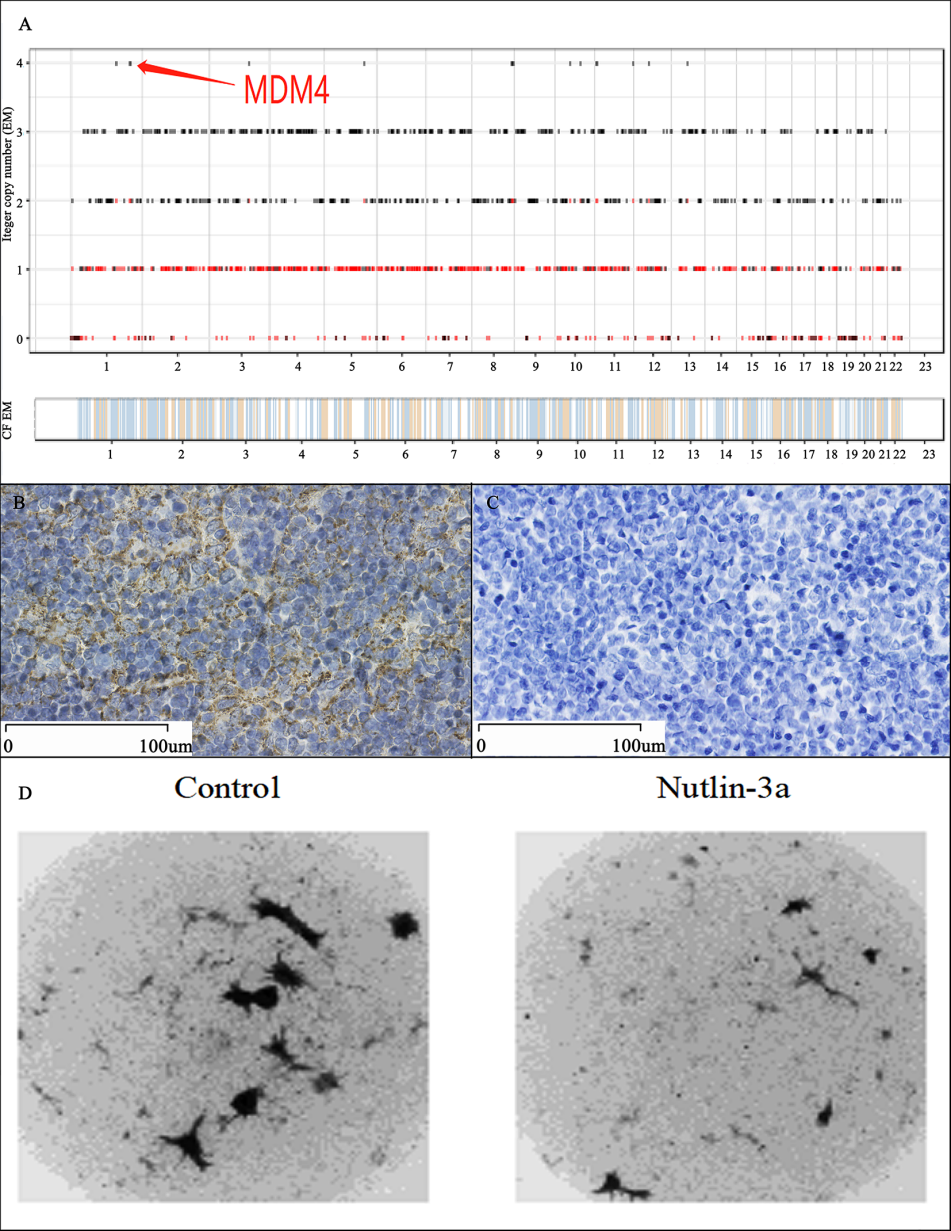
Genetic Testing and Validation Results. The WES result indicated that the MDM4 amplified in this thymoma cells, which is the unique driver gene in 99 mutated genes among 20,000 exons **(A)**; Immunohistochemical staining showed positivity for MDM4 **(B)** and negativity for p53 **(C)**; The drug sensitivity test showed that the growth rate of thymoma cells was 41.22% in Nutlin-3a group, compared with 78.66% in the control group, which meant that the thymoma cells were highly sensitive to Nutlin-3a **(D)**.

The result of WES indicated that only one driver gene-*MDM4* amplified four folds in 53.2% thymoma cells (**Figure 3A**). The immunohistochemical staining demonstrated positivity for MDM4 (**Figure 3B**) and negativity for p53 (**Figure 3C**). To further confirm the relationship between *MDM4* and tumorigenesis on thymoma, the CD-DST method was applied for testing the effect of Nutlin-3a, which is one of the MDM4 inhibitors. The growth rate of the thymoma cells is 41.22%, and it was significantly lower than 78.66% in the control group (**Figure 3D**). It meant that the thymoma cells had a high sensitivity to Nutlin-3a.

## Discussion and Conclusions

Thymoma is the most common anterior mediastinal neoplasms, originating from the epithelial cells population in the thymus. An intriguing clinical characteristic of thymoma is that the presence of thymoma is frequently associated with a variety of autoimmune diseases (4). Here we reported a thymoma patient with autoimmune enteropathy and myocarditis. This is an extremely rare case of thymoma with multiple autoimmune diseases, then cured by surgery and glucocorticoid.

Autoimmune enteropathy is a rare autoimmune disease characterized by refractory diarrhea and malnutrition. It is described primarily in infants and may occasionally occur in adults. Anti-enterocyte antibodies (AEA) and anti-goblet cell antibodies are often found in patients with autoimmune enteropathy, but not specific (6). Autoimmune myocarditis is induced by multiple factors, such as toxins, alcohols, bacteria, viruses, etc. Autoimmune myocarditis often results as idiopathic dilated cardiomyopathy, which is sometimes as a lethal disorder characterized by left ventricular (LV) enlargement and systolic dysfunction (7). In this case, preoperative examinations showed that multiple autoantibodies displayed in the patient’s serum. Remarkably, within 2 weeks after surgery, cardiac function deteriorated following the rapid decrease of LVEF. Along with the rapid exacerbation and gradual alleviation of symptoms, levels of TNI, CK-MB, LDH, and BNP substantially changed. Edrophonium testing, antibody testing and electrophysiologic testing negated the diagnosis of myasthenia gravis, despite the patient’s symptoms were similar. The clinical improvement and variations of lymphocyte subsets after tumor resection provided the evidence of the strong association between thymoma and two kinds of autoimmune diseases (autoimmune enteropathy and myocarditis).

Most interest found the *MDM4* amplified in thymoma cells by WES. IHC indicated that thymoma cells were positive for MDM4 and negative for p53 in this case, which is different from previous researches, in which we found *p53* up-regulated and *MDM4* unremarkable in thymoma (10–12). In the human genome, *MDM4* is encoded at chromosome 1q32.1 and is comprised of 11 exons (13). MDM4 expression is undetectable in most normal adult tissues (14), but less expression in spleen and thymus (15). As a p53-binding protein, Mdm4 could control p53 to regulate cell proliferation and cell cycle independently (16, 17). The relationship between *MDM4* and tumor and autoimmune diseases has been researched intensively, but no one has studied the connection between *MDM4* and thymoma and thymoma-associated autoimmune diseases. MDM4 high expression and p53 low expression may explain the particularity of this case to some extent.

Nutlins is a group of small molecules (Nutlin-1, Nutlin-2, Nutlin-3) acting by preventing the binding of MDMX to P53. Then, the accumulation of p53 activates p53 pathways, and inhibits the cancer cells growth (18). Subsequently, we used the CD-DST method to evaluate the effectiveness of Nutlin-3a in this case. CD-DST has been regarded as an effective drug sensitivity test approximating the clinical effect in different types of malignant tumors and having a high predictive accuracy for responses to chemotherapy (9). In this case, the thymoma cells were highly sensitive to Nutlin-3a. The further study confirmed that MDM4 could play an important role in the pathogenesis of this thymoma case with autoimmune enteropathy and myocarditis.

It is very important to deeply investigate the clinical characteristics and molecular abnormalities of thymoma with thymoma-associated autoimmune diseases like this case. This may provide a novel idea of pathogenesis and treatment not only for thymoma, but also for some rare autoimmune diseases. Regrettably, we cannot further confirm whether *MDM4* amplification is associated with thymoma or autoimmune diseases due to the lack of animal models.

## Data Availability Statement

The original contributions presented in the study are included in the article material. Further inquiries can be directed to the corresponding author.

## Ethics Statement

Written informed consent was obtained from the patient for publication of this case report and any accompanying images.

## Author Contributions

XD and LY conceived and designed the study. XD wrote the original draft of the manuscript. LY revised the manuscript. FL, ZY, and XGY contributed to the data curation. YJ, XTY analyzed the data. All authors contributed to the article and approved the submitted version.

## Funding

This work was supported by the Clinical Technology Innovation Project of Beijing Hospital Authority (XMLX201839).

## Conflict of Interest

The authors declare that the research was conducted in the absence of any commercial or financial relationships that could be construed as a potential conflict of interest.
